# Case report: Anorexia nervosa and unspecified restricting-type eating disorder in Jewish ultra-orthodox religious males, leading to severe physical and psychological morbidity

**DOI:** 10.3389/fpsyt.2023.966935

**Published:** 2023-04-27

**Authors:** Sofia Laufer, Estee Herman, David Serfaty, Yael Latzer, Rachel Ashkenazi, Orna Attias, Sinai Oren, Meirav Shimomi, Moria Uziel, Adi Enoch-Levy, Eliezer Witztum, Daniel Stein

**Affiliations:** ^1^Department of Eating Disorders, Maayanei Hayeshuah Medical Center, Bnei Brak, Israel; ^2^School of Social Work, Faculty of Social Welfare and Health Sciences, University of Haifa, Mount Carmel, Israel; ^3^Psychiatry Division, Rambam Health Care Campus, Eating Disorders Institution, Haifa, Israel; ^4^Eating Disorders Service for Children and Adolescents, Safra Children's Hospital, Sheba Medical Center, Tel Hashomer, Israel; ^5^Faculty of Health Sciences, Ben Gurion University of the Negev, Beer Sheva, Israel; ^6^Department of Psychiatry, Sackler Faculty of Medicine, Tel Aviv, Israel

**Keywords:** anorexia nervosa, Jewish, obsessive compulsive disorder, orthodox, religion

## Abstract

**Background:**

Young Jewish Ultra-Orthodox women usually show less disturbances in body image and eating in comparison to less religious communities. By contrast, problems with eating are highly unknown and unrecognized in Jewish Ultra-Orthodox males.

**Aim:**

To investigate whether in Ultra-Orthodox males, restricting-type AN (AN-R) with highly obsessional physical activity and unspecified restricting eating disorder (ED) in the context of obsessive–compulsive disorder (OCD) would lead to severe physical and emotional morbidity.

**Results:**

The study included two groups: the first, 3 adolescents with AN-R developing severely increased ritualized obsessional physical activity in addition to restricting eating, requiring inpatient treatment because of severe bradycardia. These youngsters ignored the severity of their obsessional physical activity, continuing with it in hospital despite their grave medical condition. One student began extensive training for triathlon, whereas another student, upon remitting from AN, developed severe muscle dysmorphia. These findings suggest that young Ultra-Orthodox males with AN may develop obsessional physical activity to increase their muscle mass rather than to lose weight Another four Jewish Ultra-Orthodox males developed malnutrition in the context of severe OCD, with no evidence of dieting or body-image disturbances. These individuals developed highly obsessional adherence to different Jewish religious rules, including prolonged praying, asceticism, and overvalued strict adherence to Jewish Kashrut rules of eating, leading in all cases to severe food restriction. They were highly unaware of their severe weight loss and required hospitalization because of severe physical disturbances associated with malnutrition. Moreover, most did not cooperate with their treatment, and their ED-related obsessionality was mostly resistant to psychopharmacotherapy.

**Conclusion:**

Owing to their highly ritualistic rigid way of life, combined with the need for excellency in studying, Jewish Ultra-Orthodox adolescent males with AN might be at a specific risk of developing severe physical disturbances if their illness is associated with highly perfectionistic obsessional physical activity. Second, Jewish Ultra-Orthodox religious males with OCD might be at a specific risk for severe undernutrition, as their rigid relentless observance of Jewish everyday laws might highly interfere with their eating.

## Introduction

Currently, anorexia nervosa (AN) is considered a genetic-related psychobiological neurodevelopmental disorder (1, 2). Nonetheless, on this biological basis, socio-cultural parameters may exert an impact on the outcome and prognosis of AN (3).

One such relevant socio-cultural aspect is religion (4). In Judaism, Ultra-Orthodox people represent the most religiously observant group, followed by Modern-Orthodox Jews, other subgroups of religious Jews (e.g., Conservative and Reform Jews), partially observant Traditional Jews, and non-religious secular Jews. Studies show that young Ultra-Orthodox women have usually (5–10), but not always (11) the most positive body image, are the least dissatisfied with their body, and show less disordered eating symptoms, followed by Modern-Orthodox women, with secular women being at the worst end of the continuum.

Considering the findings about the complex association between discorded eating and religion in Jewish religious female subgroups, the aim of the present manuscript was to describe severe restrictive eating with severe medical and emotional complications in a specific population that to the best of our knowledge has not been studied yet, i.e., Jewish Ultra-Orthodox males.

Whereas many clinical core AN-related symptoms are similar in female and male patients, several important differences do exist. Specifically, the body ideal typically presented in males centers on muscularity rather than on thinness (12, 13) or overvaluation of body weight and shape (14). Thus, in comparison to restriction which is significantly more prevalent in females, the presence of compulsive exercise is similar in male and female AN adolescents (15).

This manuscript relates to two groups of Jewish Ultra-Orthodox males with severe restricting eating disorders (EDs). The first includes adolescents with restricting-type AN (AN-R). Ultra-Orthodox adolescents leave their home around the age of 14 to study in a special boarding-school system termed Yeshiva, where they are highly invested in Holy studying. They come home only in some weekends. This may reduce eating in some vulnerable youngsters, even if unintentionally at first. As EDs are less known and suspected in Ultra-Orthodox populations, specifically in males, it might take a prolonged period before they are diagnosed.

The second group includes mostly older Ultra-Orthodox males with severe obsessive–compulsive disorder (OCD), centered around Jewish religious issues. Judaism demands strict exactness in keeping the required rules, inculcates the performance of rituals supporting it from childhood, and views their non-performance as wrong or sinful (16, 17). Rituals concerning exactness are common in Ultra-Orthodox Jews with OCD (17), but also in people with AN (18).

Research about the association between OCD and food restriction in Ultra-Orthodox males is scarce. In one of the few existing surveys (17), in a sample of 34 psychiatric outpatients with OCD from Jerusalem, Israel, religious OC symptoms were found in 13 of the 19 Ultra-Orthodox patients in this cohort, compared to only one of the 15 non-Ultra-Orthodox patients. The main religious topics identified in the Ultra-Orthodox cohort included OC symptoms related to prayer (prolonged periods of praying), dietary practices (too many food types that are not allowed to eat because of too strict religious Kashrut regulations—i.e. what is not allowed to eat in Judaism), menstrual practices, and cleanliness before praying.

## Materials and methods

### Case series

The present article describes three male patients with AN-R and four male patients with atypical restricting ED (19) resulting from severe OCD (19), all developing severe physical deterioration requiring hospitalization. Their names are not provided, and all their demographic data have been changed, to preclude their identification. Ethical review and approval is not required for this type of study in human participants in accordance with the local legislation and institutional requirement. Written informed consent to publish the manuscript has been provided by the participants and their legal guardians as required.

The first group included two Jewish Ultra-Orthodox male adolescents and one Jewish Modern-Religious male adolescent. All were diagnosed with AN-R according to the DSM 5-TR (19) criteria, with highly excessive obsessional ritualistic physical activity, exceeding their intentional food restriction. Their pre-hospitalization data is summarized in Table 1, and their admission, hospitalization, and post-hospitalization data in Table 2. Both tables include specific data for each patient.

**Table 1:** Three adolescents with DSM 5-TR AN: pre-hospitalization data.

Age and school year at admission (years)	Family background/early development/problems before AN (including weight history)	Onset of AN	Symptoms
1. 13.5, 9th grade	4/6 children; no^1^; no^2^; ⇑ weight since age 8; at age 12 BMI > 95th centile*	6–12 months before hospitalization (around age 13-Bar-Mitzva)	Restricting, intentional weight loss; mainly ⇑ obsessional physical activity – 1.5 h daily 400 lift-ups, 200 pushups
2. 17.4, 11th grade	10/10 children; no^1^; congenital ptosis of right eyelid, stuttering, school problems; ⇑ weight since age 9–10; at age 16 BMI > 95th centile*	8 months before hospitalization	School refusal since 9th grade; severe conflicts with parents about religiosity; restricting, intentional weight loss; mainly ⇑ 3 h daily physical activity; 50 kg ⇓
3. 15, 10th grade	1/3 children; no^1^; no^2^; sharp weight ⇑ in previous year; 6 months before hospitalization, after leaving home for 1st time to Yeshiva, ⇓ in weight	6 months before hospitalization	Restricting, intentional weight loss; mainly ⇑ obsessional daily physical activity—triathlon; self-prepared protein diet 800–1,000 kcal/24 h; ED-related obsessions; lost 8 kg; blackouts

DSM 5-TR: Diagnostic and Statistical Manual for the Diagnosis of Mental Disorders, fifth edition text revised (19); AN-anorexia nervosa; BMI-body mass index; no^1^: no problems in family; no^2^: no problems in early development; *according to the National Center for Health Statistics [see Centers for Disease Control and Prevention’s Year 2000 Growth Charts, adapted for Israeli children and adolescents (20)].

**Table 2:** Three adolescents with DSM 5-TR AN: admission, hospitalization and post-hospitalization data.

Admission	Hospitalization	Medications	Follow-up	Diagnosis/outcome
1. Weight < 5th centile Height > 95th centile BMI < 5th centile*; HR 35 BPM ⇑ cholesterol ⇓ T3	8 months; nasogastric tube feeding; continuing ⇑ obsessional physical activity	Fluoxetine 50 mg/day Risperidone 0.75 mg/day	Quick dropout post-hospitalization; after 1 year: weight 90%; height 50–75%; BMI > 95%*; obsessional physical activity to build muscles; self-imposed diet of 3,500 kcal (dense proteins)	AN-R ⇒ muscular dysmorphia (body building disorder)
2. Weight < 50th centile Height > 95th centile BMI < 5 centile*; hypoglycemia, ⇓ phosphor (i.e., mild refeeding syndrome), ⇓ T3; HR 40 BPM	7 months; continued ⇑ physical activity in hospital, stood for hours	No medications because of parents’ refusal	Became secular (non-religious); at discharge and follow-up: no AN preoccupations and no ⇑ physical activity	AN-R; at discharge and follow-up no ED
3. Weight 5–10th centile, height 10th centile, BMI 17.5th centile*; HR 32–35 BPM	1st hospitalization in pediatric department full cooperation, weight ⇑; 2nd hospitalization in pediatric department after 4 months because of quick relapse (800 kcal/24 h, ⇑ physical activity); HR 40 BPM, laboratory examinations OK.	Sertraline 100 mg/day Risperidone 1.5 mg/day	after 2nd hospitalization Yeshiva, continuation of ambulatory treatment for 1.5 years: supervised physical activity; at the end of school new Yeshiva far away ⇒ relapse; returned home to university studies; no AN symptoms, no ⇑ physical activity; lost interest in triathlon	AN-R; after 2–3 years no ED

DSM 5-TR: Diagnostic and Statistical Manual for the Diagnosis of Mental Disorders, fifth edition text revised (19); AN-anorexia nervosa; AN-R-anorexia nervosa restricting type; BMI-body mass index; BPM-beats per minutes; ED-eating disorder; HR-heart rate; T3-Triiodothyronine; *according to the National Center for Health Statistics [see Centers for Disease Control and Prevention’s Year 2000 Growth Charts, adapted for Israeli children and adolescents (20)].

Several important conclusions can be drawn from these tables. The three adolescents had no evidence of psychiatric family history; the developmental history before the onset of eating-related problems was normal in two cases (#1 and #3). All showed significant increase in their weight before developing intentional restriction and weight reduction. Socio-cultural triggers to weight reduction were related to normal developmental processes the Bar-Mitzva celebration occurring at age 13 for every Jewish boy (case #1) and leaving home to study in a Yeshiva (case #3).

In all cases, the highly obsessional ritualistic characteristics of the physical activity exceeded that of intentional restriction (see Table 2). They were not only for weight reduction, but also for increasing muscle mass (case #1), semi-professional sports fitness (triathlon, case #3), and to reduce stress and increase self-control in the face of severe conflicts with parents (case #2).

All three adolescents were hospitalized because of severe bradycardia. Two did not cooperate with the requirement to reduce physical activity in the hospital (cases #1,#3). Upon discharge from inpatient treatment, none had evidence of AN symptoms. In case #2, the reduction of AN symptoms, specifically of the compulsive physical activity, occurred within a relatively brief period, when his parents accepted his wish to become non-religious (he later joined the Israeli army, which is forbidden in Ultra-Orthodox communities).

For the next years, a repeated pattern was observed for case #3. Whenever returning to the Yeshiva, he started again with restriction and excessive obsessional physical activity, requiring again hospitalization because of severe bradycardia. Finally, at the age of 18, he went to a Yeshiva near his home, where the demand for excellency in studying was as perfectionistic as elsewhere, but where he could sleep at home each night and take care of his food menu. His eating stabilized, his physical activity decreased, and he had no longer interest in triathlon.

For case #1, at follow-up 1 year after discharge, his BMI was >95th percentile (see Table 2). He continued with daily strict obsessive physical activity for the sole purpose of increasing his muscle mass and planned for himself a daily diet of over 3,500 calories with mostly dense proteins. He had no core symptoms of AN, transitioning to DSM 5-TR (19) diagnosis of body dysmorphic disorder (specifier muscle dysmorphia), and functioning well in his daily living.

The second group includes one adolescent and three adult Ultra-Orthodox males with DSM 5-TR (19) OCD, with no symptoms indicative of AN, whose severe malnutrition is directly related to their OCD. Their pre-hospitalization data is summarized in Table 3, and their admission, hospitalization, and post-hospitalization data in Table 4. Several important conclusions can be drawn from these tables.

**Table 3:** One adolescent and three adults with DSM 5-TR unspecified restricting-type eating disorder resulting from OCD; pre-hospitalization data.

Age at admission (years)	Family background/early development; problems before ED	Onset of ED	Symptoms
4. 16; 11th grade	1/7 children; no^1^; no^2^; outstanding student, ⇑ expectations; tendency for ⇑ weight since childhood.	1 year before hospitalization	⇓ weight because of ⇑ in religious studying,⇑ observance of Jewish Kashrut food laws, prolonged praying; ⇓eating as did great model Torah scholars, freezing showers (⇑ asceticism); tightening belt to separate ⇓ body “lust” part, from ⇑ spiritual part; no AN symptoms
5. 31, married +4	No^1^; from early childhood rituals about food and prayers	In past 3 years ⇓ from 84 kg to 48 kg	After 18, ⇑ rituals about food and prayers, with ⇑ food restriction; from 28 ⇓ in any physical pleasure (walking to control lust and no bus to avoid contact with women); no AN symptoms
6. 39, married +6	No^1^; no^2^	During COVID-19 financial problems ⇒ ⇑ anxiety ⇒ ⇓ eating, weight, sleep and concentration	⇓ 25 kg in 3 months; no AN symptoms
7. 48, married +8	No^1^; from early age OCD, treated with Fluoxetine, Risperidone; gradually did not leave home and stopped eating because of compulsive handwashing when out/eating	Several years	⇓ 20 kg; ⇓ in eating and weight because of compulsive handwashing for hours before eating

DSM 5-TR: Diagnostic and Statistical Manual for the Diagnosis of Mental Disorders, fifth edition text revised (19); AN-anorexia nervosa; ED-eating disorder; OCD-obsessive–compulsive disorder; no^1^: no problems in family; no^2^: no problems in early development.

**Table 4:** One adolescent and three adults with DSM 5-TR unspecified restricting-type eating disorder resulting from OCD: admission, hospitalization, and post-hospitalization data.

Admission	Hospitalization	Medications	Follow-up	Diagnosis/outcome
4. weight < 6th centile; height > 85th centile; BMI < 6th centile; * HR 41 BPM day, 33 BPM night	4 months; ICU ⇒ pediatric department; in the beginning refused to eat because of Kashrut (the hospital is Jewish Ultra-Orthodox) and to suppress evil lust.	No; ⇑ in eating and weight ⇒ ⇓ in religion-related obsessions	At discharge weight 10–25 centile; height 25–50 centile; BMI 10 centile*	OCD ⇒ unspecified restricting ED (no AN symptoms); still adheres to strict religious observance in general and in eating in particular; no ⇓ in ascetic behaviors despite ⇑ in weight; still fighting with lust; no AN symptoms
5. Psychiatric department; weight 48 kg, height 1.77 m, BMI 15.4 kg/m^2^; HR 40 BPM; transferred to internal medicine department; laboratory examination: ⇑ liver enzymes, ⇓ phosphor (i.e., refeeding syndrome)	⇓ cooperation (disappeared to pray) ⇒ HR 44 BPM ⇒ ICU: ⇑ praying and physical activity and ⇓ eating to control “lust”; no AN symptoms	2nd hospitalization: Sertraline 200 mg/day Olanzapine 5 mg/day.	Discharged himself from hospital when medically stabilized against medical advice; rehospitalization after 1 month: weight 48 kg, BMI 15.3 kg/m^2^; HR 60 BPM; discharged after 3 months: weight 67.6 kg, BMI 21.5 kg/m^2^; 6 months day center: discharge weight 84 kg, BMI = 26.9 kg/m^2^ (similar to pre-illness)	Severe OCD ⇒ unspecified restricting ED (no AN symptoms); good remission of ED, partial remission of OCD
6. weight 40 kg, height 1.66 m, BMI 14.5 kg/m^2^; ⇓ glucose, phosphor (i.e., refeeding syndrome)	⇓ eating to atone for everyday sins (disclosed only during hospitalization) and because of ⇑ keeping of Kashrut laws (the hospital is Jewish Ultra-Orthodox); later in psychiatric department hid food, complained of “fullness”	Sertraline 200 mg/day, Olanzapine 7.5 mg.	After 4 months weight 45.7 kg BMI 16 kg/m^2^ again weight ⇓ when going home for weekends; discharged himself against medical advice	OCD ⇒ ⇓ eating and weight because of religious issues; allegedly some AN behaviors (food hiding, “fullness, denial of medical severity of ⇓ weight), but no AN-related obsessions
7. Weight 38 kg, height 1.70 m, BMI 13.1 kg/m^2^: ⇓ Natrium = 132 mmol/l; ⇓ albumin = 2.4 g/dl ⇓; ⇑ liver enzymes, rhabdomyolysis (⇑creatine phosphokinase (CPK) 600 u/L)	Hospitalized in internal medicine department for 3 weeks; refeeding syndrome (⇓ phosphor); nasogastric tube feeding; patient and family refusing psychiatric consultation and psychiatric hospitalization: 1. would intervene with children’s marriage prospects; 2. because problem is considered physical	Fluoxetine 60 mg/day, Olanzapine 7.5 mg/day	Discharged himself against medical advice; at discharge ⇓ Natrium, normal urea and CPK; lost to follow-up	OCD; avoiding food because of 1. “classical” handwashing compulsions—food is dirty, contaminated; 2. religious-related handwashing required in Judaism before meals, but here overly prolonged and compulsive

DSM 5-TR: Diagnostic and Statistical Manual for the Diagnosis of Mental Disorders, fifth edition text revised (19); AN, anorexia nervosa; BMI, body mass index; BPM, beats per minutes; ED, eating disorder; HR, heart rate; ICU, intensive care unit, OCD, obsessive–compulsive disorder; *according to the National Center for Health Statistics [see Centers for Disease Control and Prevention’s Year 2000 Growth Charts, adapted for Israeli children and adolescents (20)].

First, none had a psychiatric family history. Two had evidence of OCD from childhood. All three adults had a history of weight reduction for several years before hospitalization, likely indicating denial of the severity of their developing malnutrition.

The development of severe malnutrition in these four patients was primarily associated with the highly religious content of their obsessions, interfering with normal eating, e.g., excessive time devoted to praying, and faulty interpretation of the Jewish food Kashrut laws. This led to the consideration of the food cooked in the Ultra-Orthodox medical center where they were hospitalized as not Kosher enough. All demonstrated elevated OC characteristics interfering with an adaptive handling of their overvalued religious-related behaviors, including elevated rigidity, preservation, and perfectionism (21, 22). All were hospitalized with severe bradycardia, and their medical condition at admission and during hospitalization was highly compromised (see Table 4).

The greater religious observance in the adolescent patient (case #4) was related to his high aspirations and perfectionistic studying when leaving to the Yeshiva, alongside a wish to suppress almost all earthly pleasures to purify his body from urges. This led him to keep strict rituals in his praying and eating behavior, as well as to tightening of his belt to separate his upper spiritual part from his lower “lust” part, all leading, unintentionally, to severe food restriction.

During hospitalization, the patient said that he did not eat mostly to suppress all evil lust to become an outstanding Torah scholar. Later, while his weight and eating stabilized, he still adhered to strict obsessive religious observance in general and in his eating in particular, and his ascetic behaviors did not decline.

All three adult patients were in their 30th-40th when hospitalized, with severe weight reduction over a period of several years (36 kg in case #5, 25 kg in case #6, 20 kg in case #7); this indicate denial of the patients and their families of their grave physical deterioration.

Case #5 suffered from early childhood from rituals related to eating and praying. At the age of 18, he stared to considerably increase his praying, and avoided certain food types as means to identify with a famous Rabbi, known for his asceticism. Toward the birth of his third child, he decided to be stricter religiously and to abstain from any physical pleasure. Thus, he started walking instead of taking the bus, to avoid eye contact with women, and avoided sweets “to control his desire.”

The food restriction of case #6 was evident from the onset of the COVID-19 pandemic, related to financial problems. Regarding case #7, he repeatedly washed his hands for prolonged periods before eating whenever he returned from being outside. Gradually he stopped leaving his home completely. Finally, he stopped eating at all and refused to move outside his bed.

The three adult patients (and their families) were seemingly not fully aware of their grave medical condition before and during hospitalization. Case #5 often disappeared from the department against medical advice to pray at the hospital’s synagogue, leading to missing his meals. Finally, he discharged himself against medical advice, claiming that he never had any psychiatric disturbance, and all his problems were physical. When re-hospitalized 1 month later, he agreed to take medications (Sertraline 200 mg/day and Olanzapine 5 mg/day). His physical condition and eating gradually improved. His weight at his final checkup was 84.4 kg (BMI of 26.9 kg/m^2^), which was his average weight before starting with restriction.

Case #6 was diagnosed for the first time with severe OCD related to prolonged praying to atone for everyday sins only during inpatient treatment. After 4 months, when his physical condition somewhat stabilized, he discharged himself against medical advice.

For case #7, it was not clear whether he avoided food to reduce his prolonged hand washing because of regarding food as dirty and contaminated, and/or because of the requirement of Jewish religious codes of meticulous hand washing before every meal. After 3 weeks of inpatient treatment, in which he was mostly fed with a nasogastric tube (he did not need to wash his hands with nasogastric feeding), he discharged himself against medical advice.

Last, the response of the three adult to pharmacotherapy with adequate doses of SSRIs (Sertraline and Fluoxetine) and 2nd-generation antipsychotics (Olanzapine) was unfavorable (except for case #5 during his second hospitalization; see Table 4).

## Discussion

The aim of the present case series was to describe the development of AN-R and of atypical restricting-type ED (19), with severe resultant physical deterioration, requiring hospitalization, in Jewish Ultra-Orthodox males. The first group included 3 adolescents with AN-R, where one of the leading symptoms was a highly excessive obsessional physical activity. The second group included one adolescent and three adult males, whose ED resulted from severe OCD. In this group, the reduction in food intake and the subsequent deterioration of the physical state were related to highly excessive praying, overly strict food-related Kashrut keeping, and spiritual asceticism, with no evidence of AN-related stigmata.

Regarding the first group, it is of note that in contrast to secular non-religious Jewish Israeli adolescents, where diverse forms of physical activities are part of their daily routine, physical activity is not supported, and even discouraged in Ultra-Orthodox youngsters, where excellence in learning (of religious material), is not only required, but highly praised.

The question then arises as to why these three Ultra-Orthodox male adolescents arrived at a point where their “excellence” in physical activity endangered their lives. First, all experienced a considerable earlier increase in weight, when premorbid overweight is a significant risk factor for the development of AN in male adolescents (23).

Second, all were characterized with psychological traits associated with AN, including rigidity, perfectionism. Obsessionality, and perseverance (18, 24, 25), that have the potential to induce, and maintain, excessiveness, in the present case of physical activity.

Third, all left home to study in the Yeshiva, within a short period before the appearance of disturbed eating. Leaving home, in the context of severe social and academic pressure for excellency, might have been overly stressful for these youngsters.

Fourth, these adolescents developed a highly perfectionistic, ritualistic, almost “professional” physical activity plan: exercising for muscularity in case (1), highly excessive physical training in case (2), and triathlon training in case (3). It is as if the atmosphere of excellency in the Yeshiva met in these vulnerable youngsters with an unrecognized need for perfectionistic, obsessional sports activity. This became even more clear when considering that once the emotional conflicts were solved for case (2), and the living conditions were settled for case (3), their overvalued sports activity subsided. Case (1) was the only youngster continuing with obsessional physical activity, but for him, muscle development was from the beginning, the main goal.

Last, the development of restricting and excessive physical activity have ben likely unnoticed in the Yeshiva, where the focus is on studying. This when bearing in mind that AN is usually revealed much later in male vs. female adolescents (26), particularly in Ultra-Orthodox communities, where the whole problem of EDs is still largely unrecognized.

The 2nd subset comprised four Jewish Israeli Ultra-Orthodox patients with severe restriction of eating in the context of severe OCD. The resultant weight loss was severe enough to require hospitalization.

In cases #4, # 5, and #6, weight reduction resulted from prolonged obsessional praying (eating is not allowed in Judaism during praying) and from excessive observance of the Jewish Kashrut laws of eating. In case #7, it was not entirely clear whether his compulsive handwashing and resultant avoidance of food was related to regarding food as dirty and contaminated, and/or to excessive fulfillment of Jewish religious requirements.

Alongside the issues of praying and Kashrut, cases #4 and #5 reduced their eating also as an ascetic wish of control over the pleasures of life, including eating to purify their bodies from earthly pleasures and urges. The adolescent patient also took for this purpose frequent freezing showers and tightened his belt to separate his lower “lust” part, from the spiritual upper part, and case #5, stopped taking the bus to avoid eye contact with women. Reduction of eating to reduce sexual desires (fighting against the sexually invested “evil nature” in Judaism) is highly relevant in the development of disturbed eating in young males (12), specifically in Ultra-Orthodox communities. Where issues of masturbation, sex before marriage, sexual orientation and gender dysphoria are unspoken forbidden topics.

It is of note that whereas Christianity may support appetitive control and asceticism [including self-starvation for atonement (27, 28)], in Judaism, people are considered to have sinned if they willfully abstain from essential physical needs, including food (16, 29). Despite this clear requirement, case #6 has specifically reduced his eating to atone for his everyday sins. Along this line, past prominent Jewish religious figures have fasted to abolish any physical pleasure as an act of atonement for the sins of the entire community (4, 30). Some Ultra-Orthodox people nowadays, including case #4 here, may still admire these figures, identify with them, and incorporate their behaviors.

Religious Jews adhere every day to the strict codes of observance, here to those related to the manner of praying and allowed/forbidden food, but with no disturbance in their daily life. By contrast, in the individuals with OCD in the present case series, the interpretation of the Jewish regulations has been highly overvalued, leading to frank misperception of reality (e.g., you cannot stop praying to eat, and no food is Kosher enough), leading to severe deterioration in functioning and health.

To perform their payer with what they consider the required devotion, our OCD patients have prayed for many more hours than required, in some cases to metaphorically “clean their head” from forbidden thoughts. Similarly, an earlier case series described weight loss resulting from religious zeal in three young Jewish Ultra-Orthodox males. These individuals “indicated that they had restricted their diet in a misguided attempt not to “indulge” themselves, as a misinterpretation of what they had been taught in their studies in the Yeshivas” (30).

Three other factors seem of importance. Similar to the 3 adolescents with AN-R, there was no evidence of psychiatric family history also in the patients with OCD (although this could have been hidden because of stigma-related issues, specifically relevant in Ultra-Orthodox communities). Second, the three adults with OCD did not acknowledge the psychiatric origin of their weight loss or the severity of their medical condition, did not cooperate with their treatment, and actually discharged themselves against medical advice, while mostly still being underweight. Third, they did not respond well to a combination of SSRIs and 2nd-gereration antipsychotic medications.

### Summary

The present case series described the occurrence of psychologically related food restriction leading to severe weight loss with physical deterioration requiring hospitalization in Jewish Israeli Ultra-Orthodox males. Two subsets of this population were described, 3 adolescents with AN-R and one adolescent and three adults with atypical restricting-type ED (19), resulting from severe OCD, laden with highly religious content. Although the lack of psychiatric family history, the presenting ED symptoms, and the severity of the physical state were similar, the two groups were highly different. In the adolescent group, the severity of the AN symptoms was related to severe, ritualistic, highly structured and exaggerated sports activity, geared for muscularity. The message to bring home here is the need to increase the knowledge about the possible, although rare, occurrence of AN in young Ultra-Orthodox males, who leave their home to a Yeshiva for the first time.

In the mostly adult male group with severe OCD, the loss of weight has not resulted from core AN-related concerns. Rather, their ritualistic, dysfunctional religion-laden obsessionality has been embedded in their life for a prolonged period. This might explain their lack of awareness and acknowledgment of the psychiatric origin of their weight loss, and their resistance to change and to treatment. Nonetheless, it can be argued that the lack of insight of these patients into the seriousness of their illness, alongside their inability to gain weight before inpatient treatment can be potentially associated with a DSM 5 (19) diagnosis of AN.

The message to bring home here is that severe OCD should be considered in prolonged food restriction and weight loss in Ultra-Orthodox adult males. Last, this study suggests that, similarly to findings elsewhere (12, 13), the influence of religiosity on eating-related issues in Ultra-Orthodox Jewish Israeli men is highly multifaceted, contradicting the premise that greater religiosity is necessarily associated with less eating-related pathology.

## Data availability statement

The original contributions presented in the study are included in the article/supplementary material, further inquiries can be directed to the corresponding author.

## Ethics statement

Ethical review and approval was not required for the study on human participants in accordance with the local legislation and institutional requirements. Written informed consent to publish the manuscript was provided by the participants and their legal guardians/next of kin as required.

## Author contributions

SL, EH, DSe, YL, and DSt contributed the different case reports. RA, OA, SO, MS, MU, and AE-L provided important data about the different cases. DSt and EW were responsible for the organization and writing of the article. All authors contributed to the conception and design of the study, read all drafts, provided useful comments, and approved the final draft of this article.

## Conflict of interest

The authors declare that the research was conducted in the absence of any commercial or financial relationships that could be construed as a potential conflict of interest.

## Publisher’s note

All claims expressed in this article are solely those of the authors and do not necessarily represent those of their affiliated organizations, or those of the publisher, the editors and the reviewers. Any product that may be evaluated in this article, or claim that may be made by its manufacturer, is not guaranteed or endorsed by the publisher.
